# Assessing the contribution of harvested wood products under greenhouse gas estimation: accounting under the Paris Agreement and the potential for double-counting among the choice of approaches

**DOI:** 10.1186/s13021-019-0129-5

**Published:** 2019-11-26

**Authors:** Atsushi Sato, Yukihiro Nojiri

**Affiliations:** 1 Graduate School of Science and Technology, Hirosaki University, 3 Bunkyo-cho, Hirosaki, Aomori, 036-8561 Japan; 2 Mitsubishi UFJ Research and Consulting Co., Ltd., 5-11-2, Toranomon, Minato-ku, Tokyo, 105-8501 Japan; 3 Greenhouse Gas Inventory Office of Japan, Center for Global Environmental Research, National Institute for Environmental Studies, 16-2, Onogawa, Tsukuba, Ibaraki 305-8506 Japan

**Keywords:** HWP, Common approach, INDC, Global double-counting, Global no-counting, Paris Agreement

## Abstract

**Background:**

There are multiple approaches for estimating emissions and removals arising from harvested wood products (HWP) based on differences between when and where a given carbon stock change is calculated. At this moment, countries are free to use any HWP approach to prepare their annual greenhouse gas (GHG) inventory and determine emission reduction targets for their Nationally Determined Contributions (NDCs), although under the Paris Agreement (PA), the production approach is used for standard reporting in GHG inventories. Global double-counting and non-counting of HWP might occur depending on the HWP approach each country uses; however, the impact of such double-counting and non-counting has not been thoroughly evaluated.

**Results:**

We identified all cases of global double-counting and non-counting of HWP for combinations of the six HWP approaches: ‘instantaneous oxidation’, ‘stock-change’, ‘production’, ‘stock-changes approach for HWP of domestic origin (SCAD)’, ‘simple-decay’ and ‘atmospheric-flow’ approaches. In Intended Nationally Determined Contributions (INDCs), forest land is often partly or completely excluded, especially by developing countries. In such cases, HWP approaches that require comprehensive national data on wood harvesting and trade are not suitable for estimating HWP contributions. In addition, most developing countries apply the ‘instantaneous oxidation’ at the time of harvesting. Recent GHG inventories from Annex I countries show the averaged contribution of annual HWP emissions or removals to national total emissions is nearly 1%; therefore, the potential contribution of HWP to the accounted emission reduction volume is assumed to be a smaller value.

**Conclusions:**

Instantaneous oxidation remains a pragmatic approach for countries in which wood production is not a dominant part of the economy. The combination of ‘instantaneous oxidation’ with the ‘production’, ‘SCAD’ or ‘simple-decay’ approaches could be a practical solution to realize a global HWP accounting approach the eliminates double-counting. Regardless of how global double-counting and non-counting occur, the amount is not large. To improve the accuracy of the global assessment, it is important to reduce the uncertainty of estimation regarding when and how much HWP-related emissions occur at national level.

## Background

### The six HWP approaches

The carbon absorbed by trees remains in harvested wood until products made from this wood decay or are burned. Harvested wood products (HWP) contribute to carbon sequestration and the mitigation of climate change through increased use and end-of-lifecycle use of long-lived wood products, the use of by-products (wood waste) for energy, and the substitution of wood from sustainably managed forests for non-wood material in the construction sector (e.g. concrete, steel, etc.) [1, 2]. The International Panel on Climate Change (IPCC) has provided several approaches for estimating the greenhouse gas emissions and removals associated with HWP at the national level, including ‘instantaneous oxidation’ (IO), ‘stock-change’ approach (SC), ‘production’ approach (P) and ‘atmospheric-flow’ approach (AF). Each approach has different definitional system boundaries and timings of counting emissions and removals.

The Revised 1996 IPCC Guidelines, which were officially adopted by the United Nations Framework Convention on Climate Change (UNFCCC), provide the first methodological guidance for the preparation of national GHG inventories. The default approach of HWP recommended by this Guidelines is ‘instantaneous oxidation’, in which it is assumed that all of the carbon in wood is oxidized and emitted to the atmosphere when that wood is harvested and removed from the forest; this further assumes that the carbon inflow resulting from harvesting does not affect the size of the existing pool of wood products. But the IPCC itself recognized that this assumption would lead to inaccurate estimation of carbon stock changes when the size of the wood-products pool changes [3].

In the 1998 IPCC expert meeting, three other approaches (‘stock-change’, ‘production’ and ‘atmospheric-flow’) were identified and discussed [4].

The UNFCCC produced a technical paper in 2003 [5] and held a workshop on HWP in 2004 [6], leading to estimations of the potential amount of carbon stock changes for the major developed countries based on multiple HWP approaches with the discussion from various aspect. The results showed that the amount of calculated carbon stock change varied widely according to the approach used by each country and that certain approaches were beneficial for some countries (e.g., large removals were expected) but not for other countries (e.g., large emissions were expected). The advantages and disadvantages depended on which criteria were taken into account and/or how GHG emissions were estimated [4]. It was therefore difficult to agree on a single common approach regarding the incorporation of HWP in the calculation of annual national GHG inventories for use in intergovernmental negotiations, despite the fact that technical aspects of the proposed methodologies had been adequately developed over a long time.

**Fig. 1:**
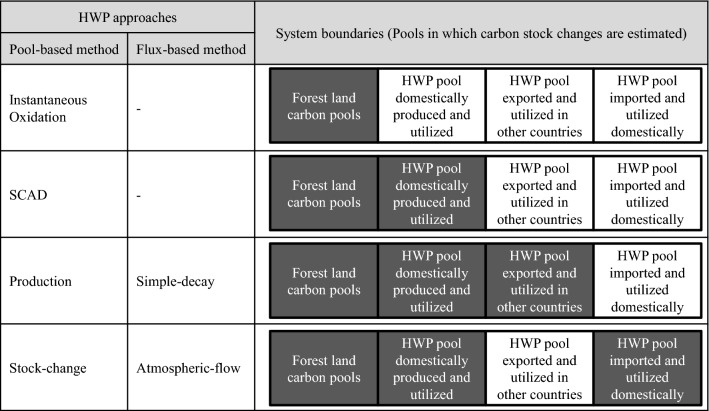
Each HWP approach includes the components with grey color and exclude the components with write color

The demand for methodological approaches to properly estimate carbon stock changes and emissions from HWP was partly reflected in subsequent IPCC methodological guidance. The Good Practice Guidance for Land Use, Land-Use Change and Forestry (GPG-LULUCF) continued using ‘instantaneous oxidation’ as the default approach but provided guidance for the application of the three other approaches in its appendix [7]. The 2006 IPCC Guidelines for National Greenhouse Gas Inventories suggested applying the instantaneous oxidation when the relevant HWP values are insignificant. The 2006 IPCC Guidelines also provided guidance for estimating emissions and removals associated with HWP based on the ‘stock-change’, ‘production’ and ‘atmospheric-flow’ approaches when HWP values are not insignificant, but did not recommend the use of any one approach over the other [8]. The 2006 IPCC Guidelines also included ‘simple-decay’ approach, which was originally suggested by Ford-Robertson [9]. This approach uses the same system boundary as the ‘production’ approach but with different terms. The 2013 Revised Supplementary Methods and Good Practice Guidance Arising from the Kyoto Protocol (2013 KPSG) [10] provided more precise methodological information on the ‘production’ based approach that was in line with the second commitment period (CP2) of the Land use, Land-Use-Change and Forestry (LULUCF) accounting rule under the Kyoto Protocol (KP) [11]. In addition to the above-mentioned HWP approaches, Cowie et al. [12] suggested the ‘SCAD’ approach. The characteristics of each HWP approach are described below. Figure 1 shows the summary of main differences of the six HWP approaches.

#### Instantaneous oxidation

Within this approach, all CO_2_ emissions and removals associated with forest harvesting and the oxidation of wood products are accounted for according to the country in which the wood was grown and the year it was harvested. Thus, all carbon contained in the HWP is accounted for as carbon loss in forest carbon pools. This is the simplest approach for estimation and reporting. This approach is applicable to various geographical boundaries from small project sites to national territories. It is possible to provide sufficient incentive to use wood based on energy and material substitution because these can reduce amount of fossil-origin energy use and biogenic CO_2_ emissions from bioenergy-use are not included in national total emissions. However, the full policy implication and the mitigation effect of HWP, including long-term use of wood products, cannot be estimated. This approach is inaccurate at the global level because there is an underlying assumption that size of the existing wood-products pool does not change, despite the fact that is estimated to increase globally over time [13]. However, ‘instantaneous oxidation’ is still widely used in estimations for GHG inventories.

#### ‘Stock-change’ approach

This approach estimates net changes in carbon stocks in forests and HWP pools through carbon gain and carbon loss. Changes in carbon stock in forests are accounted for in the country in which the wood is grown, referred to as the producing country. Changes in the HWP pool are accounted for in the country where the products are used, referred to as the consuming country. The carbon transferred from forest carbon pools to HWP pool is once accounted for as carbon loss in the forest land pool in the producing country and subsequently as carbon gain in HWP pool in the consuming countries. These stock changes are counted when and where they occur within national boundaries. In this approach, the consuming country can evaluate the policy and treatment of all wood products existing within its national boundaries [4]. This estimation method is simpler than the production approach respect to data acquisition. In terms of cross-sectoral estimation of GHG emissions from wood under the GHG inventory, the system boundaries of non-CO_2_ emissions (e.g., CH_4_ and N_2_O) from harvested wood burned in the Energy and the Waste sectors, non-CO_2_ emissions from decomposition of waste wood at solid waste disposal site (SWDS) in the Waste sector and the accounted HWP pool are the same. However, the system boundaries of forest carbon pools (domestic origin) and that of the HWP carbon pool (domestic and imported origins) are not consistent. Technically, imported wood is counted as gain of carbon in the HWP pool. This may have implications on the wood trade policies of different countries. In general, this approach is applicable to national territorial boundaries because wood transport data with respect to geographical boundaries is usually available at the national level but rarely at regional or project levels.

#### ‘Production’ approach

This approach also estimates net changes in carbon stocks in the forest and HWP pools, but attributes both to the producing country. This approach inventories domestically produced stocks only and does not provide a complete estimation of national stocks of HWP and the effect from imported wood is not evaluated. For wood products that are traded, stock changes are counted when, but not where, they occur. This approach can describe the wood lifecycle from harvesting in the forest to end-of-life. The carbon transferred from forest carbon pools to HWP pool is once accounted for as carbon loss in the forest land pools of the producing country and subsequently as carbon gain in the HWP pool of the producing country. The system boundary is the same as that of ‘instantaneous oxidation’ and is thus a trade neutral approach [4]. In terms of cross sectoral estimation of GHG emissions from wood under the GHG inventory, different system boundaries are used in the Energy and the Waste sectors (both domestically produced and imported wood are considered) and in the accounted HWP pool (only domestically produced wood is considered). But the system boundaries of forest carbon pools and HWP carbon pool are the same. Thus, this approach can be forest carbon estimation at various levels of geographical boundaries, not only national boundaries but also smaller system boundaries like projects and activities related to harvest amounts. It is also known that the calculation of domestic ratio parameter often has complexity and high uncertainty. The reporting country is responsible for carbon stocks in exported HWP even if that are not under the control of the reporting country. Obtaining explicit data on exported wood is usually difficult and leads to high calculation uncertainties.

#### ‘Stock change approach for HWP of domestic origin’: ‘SCAD’ approach

This approach also estimates net changes in carbon stocks in forest and HWP pools. This approach inventories domestically produced stocks consumed within producing country only and does not provide a complete estimation of national stocks of HWP, the effects from imported and exported wood are not evaluated [12]. This approach is often abbreviated as ‘SCAD’ approach. Changes in forest carbon stocks are accounted for in the country in which the wood is grown (the producing country). The carbon transferred from forest carbon pools to HWP pool is once accounted for as carbon loss in the forest land pool of the producing country and subsequently accounted for as carbon gain in HWP pool in the producing country but only for domestically consumed HWP. These stock changes are counted when and where they occur within national boundaries if the HWP are consumed domestically but not if they are traded. This approach is a hybrid of the stock-change and production approaches and eliminates the effect of trade as well as the uncertainty related to exported wood. The IPCC guidelines do not treat this as an independent approach. In reality, some existing reporting from the production approach does not account for the contribution of exported wood and thus the method of estimating HWP contribution becomes based inherently on this approach. The latest methodological guidelines of HWP contained in the 2019 IPCC methodological report [14] clarified how to use the terms in estimating carbon stock changes in HWP for all three pool-based approaches (‘stock-change’, ‘production’ and ‘SCAD’). As import data are not taken into account, this approach could be applicable from the national level to small project level.

#### ‘Simple-decay’ approach

This approach uses the same system boundary as the ‘production’ approach so the features arising from the system boundary are the same of those in ‘production’ approach. However, estimation by this approach focuses on the emission of carbon from forest or HWP pools to the atmosphere just like the ‘atmospheric-flow’ approach does. Thus, the carbon transfer from forest carbon pools to HWP pool is not counted as carbon loss in the forest land pools of the producing country but is counted as emissions from HWP pool at the time of end-of-life of HWP in the producing country.

#### ‘Atmospheric-flow’ approach

This approach accounts for net emissions or removals of carbon to/from the atmosphere when and where emissions and removals occur within national boundaries. Removals of carbon from the atmosphere due to forest growth are accounted for in the producing country, while emissions of carbon to the atmosphere from oxidation of harvested wood products are accounted for in the consuming country [4]. Thus, the carbon transferred from forest carbon pools to HWP pool is not accounted for as carbon loss in forest land pools in the producing country but is accounted for as emissions at the time of end-of-life of HWP in the consuming country. This approach is consistent with GHG emissions from fuel consumption and directly reflects the carbon exchange between land and the atmosphere. This approach provides incentives for not releasing emissions and for promoting wood products recycling. Like the ‘stock-change’ approach, the ‘atmospheric-flow’ approach is affected by trade and sometimes shows a huge net sink from the land use sector in countries that export large amounts of wood and wood products [6]. This approach is generally applicable for national territorial boundaries, but not for smaller system boundaries because capturing at a small scale when and where wood is burned requires precise data that are rarely available at such a small scale.

### The treatment to date of HWP estimation under various UNFCCC schemes

There are several UNFCCC schemes relating to GHG estimation and reporting in the LULUCF sector, including the GHG inventory reporting schemes under the convention for Annex I parties (developed countries) and for non-Annex I parties (developing countries), the reporting of accounted LULUCF activities under the KP, and the reporting of Reducing emissions from deforestation and forest degradation and the role of conservation, sustainable management of forests and enhancement of forest carbon stocks in developing countries (REDD+). The treatment of HWP is slightly different in each existing scheme under UNFCCC, a summary of which is presented in Table 1.

**Table 1:** Summary of HWP treatment under various UNFCCC schemes

Scheme	HWP approach	Applied IPCC guidelines
GHG inventory before PA		
For Annex I	Production approach, stock-change approach, atmospheric-flow approach Simple-decay approach	2006 IPCC guidelines
For non-Annex I	No specific rule	Revised 1996 guidelines^a^
KP		
First commitment period	Instantaneous oxidation	GPG-LULUCF
Second commitment period	Production-based approach/instantaneous oxidation	2006 IPCC Guidelines 2013 KPSG
REDD+	No specific rule	Most recent IPCC guidelines
PA		
GHG inventory	Production approach (or instantaneous oxidation)—as common information Any approach for national GHG inventory estimation	2006 IPCC Guidelines and any subsequent IPCC guidelines
NDC accounting	Any approach	IPCC guidance (= all IPCC guidelines and guidance)

^a^Using the Revised 1996 Guidance is mandatory but using Good Practice Guidance for LULUCF is encouraged. Using the 2006 IPCC guidelines is allowed

The UNFCCC negotiation has not reached an agreement on a common approach to the GHG inventory reporting scheme under the convention for Annex I parties so all HWP approaches provided in the 2006 IPCC guidelines may be used. Furthermore, no hierarchy among approaches has been established [8, 15]. No specific guidance on HWP is given to the GHG inventory reporting scheme of under the convention for non-Annex I parties [16, 17]. In reality, those parties using the Revised 1996 IPCC Guidelines or GHG-LULUCF have applied the instantaneous oxidation. It is also noted that some non-Annex I parties have already applied the 2006 IPCC Guidelines to prepare their GHG inventory and to estimate the related contribution of HWP.

For KP-LULUCF activities [e.g., Forest Management (FM), Afforestation and Reforestation (AR) and Deforestation (D)], ‘instantaneous oxidation’ was applied for the first commitment period (CP1) [18] and either the ‘instantaneous oxidation’ or the ‘production’ approach with special rules (e.g. applying instantaneous oxidation for HWPs from deforestation and eliminating carbon stocks in SWDS) was applied for CP2 [10, 11]. The change in HWP treatment between CP1 and CP2 was due to the growing demand of parties who evaluated the mitigation effects of HWP. Thus, modifying the treatment of HWP using approaches other than ‘instantaneous oxidation’ was identified as a potential amendment for the UNFCCC negotiations concerning CP2 [19, 20]. The main reason for adopting the ‘production’ approach with special rules for CP2 was to take into account situations in which only wood products from forests in Annex I countries under the KP are included in the accounting under the scheme of the KP, meaning that wood products from forests in other countries must be excluded from the accounting. In this case, the ‘production’ approach linked to harvesting from KP-LULUCF activities in producing countries was the approach deemed most acceptable and easiest for estimation.

REDD+ provides no specific guidance on accounting for HWP and so ‘instantaneous oxidation’ is generally used when trees are harvested. This is because the methodological priority of REDD+ was set to accurately capture the carbon losses of forest pools due to deforestation and forest degradation as accurately as possible by using a combination of remote sensing techniques and ground surveys. In addition, REDD+ accounting is implemented based on reference levels. This means countries should not only estimate recent emissions and removals but also make future projections (i.e., reference levels) taking into account historical data for carbon pools that countries want to include. Therefore, the inclusion of HWP necessitates new additional data sets and projection methodologies that may be difficult to implement. However, extending utilization to include extracted timber may contribute to the overall climate change mitigation benefits from the forestry sector [21].

### Treatment of HWP under the Paris Agreement

The PA, a legal international framework for tacking climate change for the period starting after the year 2020, was adopted by the Conference of the Parties (COP) at 21st session of the UNFCCC held in Paris in December 2015 by decision 1/CP.21 [22]. Under the PA, the global GHG reporting and accounting scheme will expand to include more countries as well as more complete sources and sinks of GHG. Meanwhile, the emission reduction target for each country is not decided on the bases of a top-down decision by the UNFCCC negotiation but rather by the individual countries themselves. Each country’s NDC, which includes its emission reduction target, is communicated to the UNFCCC. INDCs of 165 countries and regional group have been communicated to the UNFCCC secretariat (the last submission of INDC was made in April 2017) [23].

The rules and modalities necessary for the PA to succeed have been discussed since COP21 and were adopted at COP24 in Katowice, Poland in December 2018. HWP approaches are mentioned in two places, in the accounting guidance for NDC mitigation outcomes [24] and in the guidance for GHG inventory reporting in modalities, procedures, and guidelines (MPGs) under the transparency framework [25].

In the accounting guidance for NDC mitigation outcomes, parties are requested to clarify which HWP approach is used when accounting for emissions and removals from HWP. This means that parties are free to choose any HWP approach for their NDC. In the guidance for GHG inventory, when emissions and removals from HWP are estimated using an approach other than ‘production’ approach, the party is requested to also provide supplementary information on emissions and removals from HWP using the ‘production’ approach. This means that parties are free to choose any HWP approach for estimating their national total emissions, however, an estimation based on the ‘production’ approach must be reported as an additional information item.

The outcome above is considered a practical solution for achieving multiple aims related to HWP reporting and accounting by allowing the aggregation of HWP contributions by each country without double-counting, as well as letting parties choose how to estimate the HWP contribution of their national total emissions. However, the risk of global double-counting of mitigation efforts among parties remains because the combination of HWP approaches taken by different countries will count the same carbon contained the HWP traded between these countries. It should be noted that Article 4.13 of the PA states that parties shall ensure the avoidance of double-counting when they account for anthropogenic emissions and removals corresponding to their NDCs; however, the primary reason for including this language was to avoid double-counting of mitigation outcome between parties once international transfer of mitigation outcomes between parties (i.e., emission trading) begins.

Thus, potential global double-counting and non-counting might occur as a result of using different HWP approaches among countries and the potential significance of such an outcome is still considered worthy clarification. In this article, three aspects of this issue are considered. The first is an assessment of the applicability of HWP approaches in submitted INDCs according to type. The second is a logical assessment of the occurrence of global double-counting and non-counting of carbon according to choices of HWP approaches. The third is an assessment of the potential contribution of HWP to estimating GHG emissions and removals and the accounting for emission reductions.

## Results

### Analysis of INDC in terms of HWP treatment and the applicability of HWP approaches for each INDC

The treatment of LULUCF or forest in INDCs is the basic information to consider the treatment of HWP. Forsell et al. [26] analyzed the treatment of the LULUCF sector in INDCs submitted by the end of 2015 (five more submissions were made after this). This analysis provided the four-broad categorization of the treatment of LULUCF in the mitigation component, (a) including LULUCF with quantifiable details (38 countries), (b) including LULUCF without quantifiable details (78 countries), (c) not including LULUCF but the final decision will be made in the future (15 countries) and (d) LULUCF is not mentioned at all (39 countries). Assessing the coverage of sector and the target type are the starting point of the analysis of NDCs and further classification is necessary depending on the aim. The estimation of emissions and removals associated with HWP is possible only when forest land is included as a GHG contribution in the INDC. In addition, the coverage of forest land and/or the amount of wood harvested in a country affects the applicability of the various HWP approaches because some HWP approaches requiring comprehensive national level data. Therefore, the INDC classification is made based on the following four factors: (1) whether or not the INDC includes forest land or not, (2) whether or not a forest-related INDC is expressed as GHG emissions/removals, (3) whether or not all forest harvesting is considered or could be included, and (4) whether or not the applied IPCC guidelines allow calculation of the HWP contribution by approaches other than ‘instantaneous oxidation’.

The abovementioned factors are detailed in Table 2 along with the global share of wood harvesting volume for each INDC classification, based on data for 2017 from the Food and Agriculture Organization’s Corporate Statistical Database (FAOSTAT) [27]. Fifty-one countries considered or could include all harvesting from forests in their INDC and thus their HWP contributions could be estimated by the methodologies provided in the 2006 IPCC Guidelines (Case A); their global share of total round wood production was 60.3%. Fourteen countries included all harvesting from forests in their INDCs but used only the instantaneous oxidation approach based on the Revised 1996 IPCC Guidelines or GPG-LULUCF (Case B); these countries accounted for 2.2% of the global share of round wood production. Twenty-eight countries included forest land in their INDCs, but forest harvesting was not completely covered in their INDC (e.g., REDD+ was used in the INDC but only deforestation was included in its REDD+) (Case C); their global share of round wood production was 18.3%. Nineteen countries included forests in their INDCs but the contribution of forests to their total GHG amount could not be quantified (Case D); their global share of round wood production was 14.0%. Fifty-three countries did not include forest land at all in their INDCs (Case E); their global share of round wood production was 5.0%. Three countries did not submit INDCs (Case F); their global share of round wood production was 0.2%.

**Table 2:** The treatment of forest, harvesting and HWP in the INDCs

INDC includes forest	Forest contribution is expressed as GHG	All wood harvesting is considered/could be included	HWP contribution is considered/could be estimated	Number of countries	The share of wood harvesting volume in 2017 based on FASTAT (%)	Countries
Yes	Yes	Yes	Yes	51	60.3	Case A: EU countries (including Croatia, Cyprus, Malta), Australia, Canada, Iceland, Japan, New Zealand, Norway, Switzerland, Turkey, Ukraine, USA, Andorra, Brazil, Brunei, Burundi, Chad, Columbia, Comoros, Congo, Costa Rica, DPR Korea, DRC, Dominica, Dominican Republic, Ecuador, Equatorial Grenada, Ethiopia, Kazakhstan, Kenya, Mali, Mexico, Namibia, Niger, Papua New Guinea, Philippines, R-Moldova, San Marino, Senegal, Serbia, Singapore, Solomon Island, South Africa, Tajikistan, Togo, Uganda, Tanzania, Vietnam, Zambia
			No	14	2.2	Case B: Angola, Argentina, Azerbaijan, Bosnia & Herzegovina, Cambodia, Guinea, Kyrgyzstan, Lebanon, Liechtenstein, Madagascar, Monaco, Paraguay, Peru, Timor
		No	–	28	18.3	Case C: Algeria, Armenia, Bahamas, Belize, Benin, Burkina Faso, Central Africa, Cote d'Ivoire, Gabon, Gambia, Ghana, Guatemala, Haiti, Honduras, India, Indonesia, Jordan, Malawi, Malaysia, Mauritius, Morocco, Mozambique, Panama, Sierra Leone, South Sudan, Tunisia, Uruguay, Venezuela
	No	–	–	19	14.0	Case D: Bhutan, Bolivia, Carbo Verde, Chili, China, El Salvador, Eritrea, Guinea Bissau, Guyana, Lao, Lesotho, Mauritania, Myanmar, Nepal, Rwanda, Somalia, Sri Lanka, Sudan, Suriname
No	–	–	–	53	5.0	Case E: Afghanistan, Albania, Antigua & Barbuda, Bahrain, Bangladesh, Barbados, Belarus, Botswana, Cabo Verde, Cameroon, Cuba, Djibouti, Egypt, Eswatini, Fiji, Georgia, Iran, Iraq, Israel, Jamaica, Kiribati, Kuwait, Liberia, Maldives, Micronesia, Mongolia, Montenegro, Nauru, Niue, Oman, Pakistan, Palau, Qatar, South Korea, Macedonia, Marshall Islands, St. Lucia, St Vincent Grenadine, Samoa, Sao Tome Principe, Saudi Arabia, Seychelles, St. Kitts and Nevis, Thailand, Tonga, Trinidad & Tobago, Turkmenistan, Tuvalu, UAE, Uzbekistan, Vanuatu, Yemen, Zimbabwe
No INDC submission				3	0.2	Case F: Libya, Nicaragua, Syrian Arab Republic

We note the classification of INDC type is almost consistent between this analysis and the analysis in Forsell et al. [26] but some countries are considered different way. It is noted that the types of INDCs and amount of information explaining those contributions in INDCs varies widely and in some case the information provided is ambiguous and lacking detail.

### Logical analysis of double- or non-counting among HWP accounting approaches

To clarify the occurrence of double-counting or non-counting of emissions or removals associated with HWP based on the accounting approaches selected by countries, we classified each approach in terms of carbon flows.

**Fig. 2:**
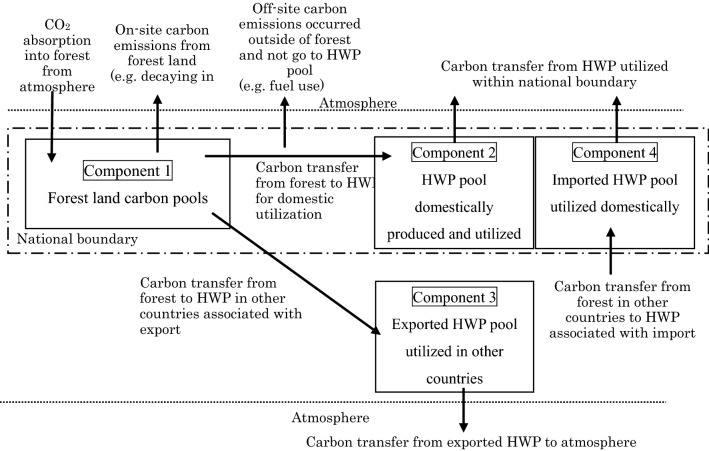
Overview of carbon flows to be considered for each HWP approach. This figure shows the fate of carbon absorbed in forest. Most of carbon returns to the atmosphere as CO_2_ but sometimes as CH_4_ or other gases due to decomposition or incineration. The type of GHG gas is not differentiated in this figure

The various HWP approaches can be simplified and summarized according to differences in how they calculate the following four components: (1) the pool of forest land, (2) the pool of domestically produced and domestically utilized HWP, (3) the pool of HWP exported and utilized in other countries, and (4) the pool of HWP imported from other countries and utilized domestically. Figure 2 shows an overview of carbon transfers for the four components and the atmosphere. Emissions and removals are estimated based on the balance of carbon inflow (carbon transfer from the outside to the target component) and carbon outflow (carbon transfer from the target component to the outside) for each component and the atmosphere.

Table 3 shows how carbon transfers are treated in each of the six HWP approaches. Within this table, the terms “FL C gain” and “FL C loss” are referred to an estimated carbon stock gain and loss, respectively, in the forest land carbon pool. Similarly, “HWP C gain” and “HWP C loss” are referred to an estimated carbon gain and loss, respectively, in the HWP pool. Differences between the HWP approaches are the result of (1) how carbon transfers from the forest carbon pool to the HWP pool are treated and (2) which HWP components are (or are not) accounted for.

**Table 3:** Treatment of carbon inflows and outflows in forest and HWP pools in each HWP approach

Carbon transfers	Pool-based approaches				Flux-based approaches	
	IO	SC	P	SCAD	Simple	AF
On-site absorption	FL C gain	FL C gain	FL C gain	FL C gain	FL C gain	FL C gain
On-site emissions	FL C loss	FL C loss	FL C loss	FL C loss	FL C loss	FL C loss
Off-site emissions	FL C loss	FL C loss	FL C loss	FL C loss	FL C loss	FL C loss
from FL to HWP as DU	FL C loss	FL C loss HWP C gain	FL C loss HWP C gain	FL C loss HWP C gain	–	–
from FL to exported HWP	FL C loss	FL C loss	FL C loss HWP C gain	FL C loss	–	–
from FL (in other countries) to imported HWP	–	HWP C gain	–	–	–	–
from HWP as DU to atmosphere	–	HWP C loss	HWP C loss	HWP C loss	HWP C loss	HWP C loss
from exported HWP to atmosphere	–	–	HWP C loss	–	HWP C loss	–
from imported HWP to atmosphere	–	HWP C loss	–	–	–	HWP C loss

*IO* instantaneous oxidation, *SC* stock-change approach, *P* production approach, *SCAD* stock change approach for HWP of domestic origin, *Simple* simple-decay approach, *AF* atmospheric-flow approach, *FL* forest land, *C* carbon, *DU* domestically utilized wood

In IO, SC, P and SCAD approaches, carbon transfer from the forest carbon pool to the HWP pool is accounted for as carbon loss in the forest land pool. For S, P and SCAD approaches this carbon transfer from the forest carbon pool to the HWP pool is again accounted as carbon gain in the HWP pool at the same time. In this approach, which is often referred to as the “pool-based approach”, emissions and removals are estimated based on carbon stock changes in forest land pools and HWP pool. For AF and Simple approaches, this carbon transfer is not accounted for either as emissions or removals, reflecting a situation in which the relevant carbon is not actually released into the atmosphere. This approach, which is often referred to as the “flux-based approach”, emissions and removals are estimated based on direct exchange of carbon flux between forest land and HWP pools and the atmosphere.

The classification in Table 3 explicitly indicates that ‘SCAD’ and Simple approaches have feature that the other four approaches including IO, SC, P and AF approaches do not. Thus, the occurrence of double-counting and non-counting should be considered in the all combination of these “six” approaches.

The occurrence of double-counting or non-counting of HWP is assessed in focusing on the carbon in traded wood from export country to import country. Table 4 provides an overview of how the carbon in traded wood is accounted for in the components of exporting country’s forest land pools, exporting country’s HWP pool and importing country’s HWP pool under all combinations of HWP approaches may selected by exporting country and importing country. “X” in Table 4 means C gain or loss is accounted for in each component under the combination of HWP approaches. For example, if exporting country selects ‘instantaneous oxidation’ and importing country selects ‘stock-change’ approach (the cased of second row from the top in Table 4), carbon in traded wood is firstly accounted for as carbon gain in exporting country’s forest land pools when it absorbed (shown as “X” in FL pools in exporting country, gain) and then accounted for as carbon loss of exporting country’s forest land pool when it goes to export (shown as “X” in FL pools in exporting country, loss). This carbon is not accounted for in HWP pool in exporting country anymore under ‘instantaneous oxidation’, therefore no “X” is showed in exporting country’s HWP pool component. While carbon in imported wood is accounted for as HWP carbon gain under ‘stock-change’ approach, “X” is showed in “HWP pool in importing country, gain”. When this carbon reaches the end-of-life of HWP and finally emitted to the atmosphere, this emission is accounted for as “HWP pool in importing country, loss” (shown as “X” to the corresponding cell).

**Table 4:** Occurrence of double-counting or non-counting in each combination of different HWP approaches

Exporting country	Importing country	FL pools in export country		HWP pool in export country		HWP pool in import country		Numbers of carbon counted		Occurrence of double-counting or no-counting		
		Gain	Loss	Gain	Loss	Gain	Loss	Gain	Loss	With imbalanced double-counting	With imbalanced non-counting	With balanced transfers but double-counting
IO	IO	X	X					1	1	–	–	–
IO	SC	X	X			X	X	2	2	–	–	–
IO	P (all)	X	X					1	1	–	–	–
IO	AF	X	X				X	1	2	Y	–	–
SC	IO	X	X					1	1	–	–	–
SC	SC	X	X			X	X	2	2	–	–	–
SC	P (all)	X	X					1	1	–	–	–
SC	AF	X	X				X	1	2	Y	–	–
P	IO	X	X	X	X			2	2	–	–	–
P	SC	X	X	X	X	X	X	3	3	–	–	Y
P	P (all)	X	X	X	X			2	2	–	–	–
P	AF	X	X	X	X		X	2	3	Y	–	–
P-SCAD	IO	X	X					1	1	–	–	–
P-SCAD	SC	X	X			X	X	2	2	–	–	–
P-SCAD	P (all)	X	X					1	1	–	–	–
P-SCAD	AF	X	X				X	1	2	Y	–	–
P-Simple	IO	X			X			1	1	–	–	–
P-Simple	SC	X			X	X	X	2	2	–	–	–
P-Simple	P (all)	X			X			1	1	–	–	–
P-Simple	AF	X			X		X	1	2	Y	–	–
AF	IO	X						1	0	–	Y	–
AF	SC	X				X	X	2	1	–	Y	–
AF	P (all)	X						1	0	–	Y	–
AF	AF	X					X	1	1	–	–	–

*X* carbon gain or loss is accounted, *Y* double-counting or non-counting exists, *IO* instantaneous oxidation, *SC* stock-change, *P* production approach, *P-SCAD* stock change approach for HWP of domestic origin, *P-Simple* simple-decay approach, *P (all)* production based approaches including P, SCAD and simple-decay, *AF* atmospheric-flow approach

When the carbon in traded wood is properly accounted for without double-counting or non-counting, the following two conditions must be met: (1) carbon gains or losses are represented by numbers 1 or 2 (We only have two pools of forest and HWP, accounting gains or losses three times means duplication occurs) (2) the numbers of gains and losses are the same (This means both gain and loss are counted in balanced way. Otherwise, unbalanced counting of gain or loss occurs).

Double-counting or non-counting of carbon occurs (1) when a combination of ‘atmospheric-flow’ and pool-based approaches is used, and (2) when wood is exported from a country using the ‘production’ approach to a country using the ‘stock-change’ approach. Additional conditions are as follows.

#### Double-counting

- Emission of carbon in traded wood from a country using ‘instantaneous oxidation’ to a country using ‘atmospheric-flow’ approach.
- Emission of carbon in traded wood from a country using ‘stock-change’ approach to a country using ‘atmospheric-flow’ approach.
- Emission of carbon in traded wood from a country using ‘production’ based approaches (including SCAD and ‘simple-decay’) to a country using ‘atmospheric-flow’ approach.
- Carbon stock change in traded wood from a country using ‘production’ approach to a country using ‘stock-change’ approach.
- Removal of carbon in traded wood from a country using ‘atmospheric-flow’ approach to a country using ‘stock-change’ approach.

#### Non-counting

- Emission of carbon in traded wood from a country using ‘atmospheric-flow’ approach to a country using ‘instantaneous oxidation’.
- Emission of carbon in traded wood from a country using ‘atmospheric-flow’ approach to a country using ‘production’ based approaches (including SCAD and ‘simple-decay’).

### Analysis of the current contribution of HWP in the reported GHG inventory

Table 5 presents a summary of HWP estimations used in the 2018 GHG inventories submitted by Annex I countries for the period from 1990 to 2016 [28]. Thirty-eight countries estimated emissions and removals arising from HWP and 5 did not.

**Table 5:** Summary of HWP reporting in GHG inventories 1990–2016 for each Annex I country

Countries	HWP approaches	HWP C stock change trend	HWP_CSC/(FL_CSC + HWP_CSC)	Net CO_2_ from HWP/national total GHG emissions (excl./incl. LULUCF)	Hypothesis CO_2_ credit of HWP (top 1/3—average)/national total net GHG emissions^a^
Australia	SC	Continuous C gains	28.6%	− 1.3%/− 1.1%	− 0.2%
Austria	P	Continuous C gains	50.7%	− 3.1%/− 3.6%	− 1.7%
Belgium	P(KP)	Total C loss	− 11.9%	0.2%/0.2%	− 0.1%
Bulgaria	P(KP)	Total C loss	− 1.7%	0.3%/0.4%	− 0.6%
Belarus	NE	–	0%	0%	0%
Canada	S	Total C gain	0.1%	0.0%/0.0%	0.0%
Switzerland	P(KP)	Continuous C gains	− 36.0%	− 0.9%/−0.9%	− 0.5%
Cyprus	P	Total C loss	41.5%	0.2%/0.2%	− 0.2%
Czechia	SCAD(KP)	Total C gain	31.1%	− 0.7%/− 0.7%	− 0.6%
Germany	P	Total C gain	16.6%	− 0.5%/−0.5%	− 0.5%
Denmark	P	Total C gain	− 26.9%	0.0%/0.0%	− 0.2%
Spain	P	Continuous C gains	5.9%	−0.6%/− 0.6%	− 0.4%
Estonia	P(KP)	Total C gain	29.3%	− 3.1%/− 3.6%	− 2.0%
Finland	P	Total C gain	12.4%	− 5.1%/− 7.7%	− 4.7%
France	P	Continuous C gains	6.1%	− 0.8%/− 0.8%	− 0.3%
UK	P	Continuous C gains	12.2%	− 0.3%/− 0.3%	− 0.1%
Greece	P	Continuous C gains	10.5%	− 0.2%/− 0.2%	− 0.2%
Croatia	SCAD(KP)	Total C gain	3.1%	− 0.8%/1.0%	− 1.3%
Hungary	P	Total C loss	− 0.6%	0.0%/0.0%	− 0.2%
Ireland	P	Continuous C gains	26.3%	− 1.3%/1.2%	− 0.4%
Iceland	SCAD(KP)	Total C loss	− 0.1%	0.0%/0.0%	0.0%
Italia	P(KP)	Total C gain	1.4%	− 0.1%/0.1%	− 0.1%
Japan	P(KP)	Total C loss	− 0.7%	0.0%/0.1%	− 0.1%
Kazakhstan	IO	–	0%	0%	0%
Lichtenstein	P(KP)	Total C gain	13.4%	− 0.3%/− 0.3%	− 0.5%
Lithuania	P	Continuous C gains	13.5%	− 4.7%/− 6.6%	− 2.0%
Luxemburg	IO(KP)	–	0%	0%	0%
Latvia	P(KP)	Total C gain	33.8%	− 12.9%/− 49.3%	− 12.4%
Monaco	NO	–	–	–	–
Malta	NE	–	0%	0%	0%
Netherland	P(KP)	Total C loss	− 3.6%	0.0%/0.0%	0.0%
Norway	P	Total C gain	2.2%	− 0.6%/− 0.9%	− 1.5%
New Zealand	P	Continuous C gains	20.4%	− 6.9%/− 11.2%	− 3.6%
Poland	P(KP)	Continuous C gains	8.7%	− 0.6%/− 0.7%	− 0.3%
Portugal	P	Total C gain	7.9%	− 1.1%/− 1.1%	− 0.6%
Romania	P	Total C gain	9.3%	− 1.7%/− 2.1%	− 2.2%
Russia	P	Total C loss	− 0.3%	0.1%/0.1%	− 0.2%
Slovakia	SCAD (KP)	Total C gain	16.3%	− 1.6%/− 2.0%	− 1.8%
Slovenia	P	Total C gain	1.3%	− 0.4%/− 0.6%	− 0.7%
Sweden	P	Continuous C gains	21.8%	− 11.0%/− 30.6%	− 7.8%
Turkey	P	Continuous C gains	11.9%	− 1.1%/− 1.2%	− 1.1%
Ukraine	P	Total C loss	− 2.4%	0.3%/0.3%	− 0.3%
United States of America	P	Continuous C gains	23.6%	− 1.4%/− 1.6%	− 0.4%
Total	–	C gains	9.2%	− 0.9%/− 1.0%	− 0.4%

*IO* instantaneous oxidation, *SC* stock-change, *P* production, *SCAD* stock change approach for HWP of domestic origin, *S* simple-decay, *KP* applying the LULUCF accounting rule for the second commitment period of the Kyoto Protocol, *NE* not estimated, *NO* not occurring

^a^Difference between a hypothetical baseline based on the average of annual carbon stock changes for the period from 1990 to 2016 and the largest carbon gains within the top one-third for the same period

Regarding the choice of HWP approaches,one country (Australia) used ‘stock-change’ approach,^1^ one country (Canada) used simple-decay type of estimation, and the other 36 countries used some type of production based approaches, among which, 21 used pure ‘production’ approach without any modification and 11 used ‘production’ approach with the KP CP2 accounting rule (i.e., wood from deforestation was estimated based on instantaneous oxidation), and 4 countries (Czechia, Croatia, Slovakia and Iceland) used ‘SCAD’ approach with the KP CP2 accounting rules.

HWP contributions from 1990 to 2016 were estimated as net removals for 29 countries and net emissions for 9 countries (indicated as “total C loss” in Table 5). HWP pools were estimated to be continuous carbon gains for all the period in 18 countries (indicated as “continuous C gain” in Table 5); the annual estimates of the other countries included both gains and losses of carbon (indicated as “total C gain” in Table 5). No country estimated its HWP pool as continuous loss of carbon for the entirety of the period. Considering all Annex-I countries as a whole, HWP pools acted as sinks or removals for the period from 1990 to 2016, which is in line with previous observations of an increase in HWP volume [13, 15].

From 1990 to 2016, the average amount of the net carbon stock changes in HWP pool in forest land carbon pools ranged from − 36 to 50% of average amount of the net carbon stock changes in forest land pools in Annex I countries (average 9.2%). This result is fairly consistent with the finding in other studies that HWP acts as a 10% contribution [30], however, it should be noted that the carbon pools included in forest land estimation are different among Annex I countries and so the above-mentioned comparison may not be fully consistent in each country level. The HWP contribution to total national GHG emissions from 1990 to 2016 is nearly 1% of the off-set level (0.9% of the emissions without LULUCF and 1.0% of the emissions with LULUCF). The HWP contribution to the total national emissions of each country was calculated to be within the range of − 12.9% to 0.3% (without LULUCF) or − 49.3% to 0.4% (with LULUCF). Johnston and Radeloff [31] provided similar values of off-set level the global emissions and concluded carbon stored within end-use HWPs varies widely across countries and depends on evolving market forces. Johnston and Radeloff [31] also evaluated there is a considerable sequestration gap (71 Mt of CO_2_e year^−1^ of unaccounted carbon storage in 2015) under the current GHG inventory reporting. As an example of a large wood-consuming non-Annex I country, China whose INDC covers forests as a non-GHG-type target, compensates approximately 2.9% of the its CO_2_ emissions from energy consumption by HWP contribution based on a research-level estimation [32], although GHG inventory has not included HWP estimation.

The potential impact of HWP in the context of accounting for emission reductions is assumed from a comparison between a hypothetical baseline based on the average of annual carbon stock changes from 1990 to 2016 and the largest carbon gains within the top one-third of countries in the same period. In this hypothetical calculation, the emission reductions archived from the HWP pool appears to be less than 0.5% of the total national emissions for nearly half of the countries but may represent a relatively large contribution (greater than 1%) for nearly one fourth of the countries (Table 5). It should also be noted that the inter-annual variability is relatively large for the HWP pool because the carbon stock change in the HWP pool is a result of the balance of inflow and outflow, both of which have their own inter-annual variability which leads to complex annual changes in carbon stocks. This situation may have implications for the way the reference level/baseline is established, largely affecting how much the accounted HWP contributes to the emission reduction target.

## Discussion

### Avoiding global double-counting or non-counting in HWP with respect to GHG emissions and removals estimation

It is a given that double-counting or non-counting of carbon from traded wood would not be an issue if every country used the same HWP approach. However, it should be noted that forestry and HWP are not significant sources of emissions or sinks of removals for some countries and thus using ‘instantaneous oxidation’ is pragmatic for these countries. As such, ‘atmospheric-flow’ approach is not suitable for estimating the HWP contribution because global double-counting and non-counting can occur when a combination of ‘instantaneous oxidation’ and ‘atmospheric-flow’ approach is used.

All pool-based approaches avoid global double-counting and non-counting when used together with ‘instantaneous oxidation’. The system boundary of the production-based approaches is the same as that of ‘instantaneous oxidation’, so ‘production’, ‘SCAD’ and ‘simple-decay’ approaches can avoid global double-counting and non-counting when used together with ‘instantaneous oxidation’. However, double-counting of carbon will occur when wood is exported from a country using ‘production’ approach to a country using ‘stock-change’ approach and should therefore be avoided.

In summary, the solution for avoiding global double-counting and non-counting when some countries uses ‘instantaneous oxidation’, is for the other countries to use (1) ‘production’ approach uniformly, (2) ‘stock-change’ approach uniformly, (3) ‘SCAD’ approach uniformly, or (4) ‘production’ or ‘stock-change’ or ‘SCAD’ approach freely, but when wood is exported from a county using ‘production’ approach to a country using ‘stock-change’ approach, the double-counting is avoided by applying a special treatment only for the carbon in this traded wood in which a exporting country uses SCAD approach or a importing country eliminate carbon inflow from this traded wood products.

Under the current GHG inventory reporting, only the HWP imported to Australia, which applies the stock-change approach, from developed countries using the production-based approaches are double-counted. Australia reported the amount of imported sawn wood and wood panel as 1.2 million m^3^ as an annual average for the years from 1990 to 2016 in the Australian GHG inventory [28]. This is almost 16% of sawn wood and wood panel consumed in Australia in this period and is not large when it is compared with the total sawn wood and wood panel consumption in the world (more than 800 million m^3^ [27]). Therefore, the impact of double-counting of carbon in HWP is considered nearly negligible. In the future, the decisions of HWP approach used in some major wood-producing and wood-consuming developing countries (e.g., China, India, Chile, Indonesia and Malaysia) must become important for avoiding global double-counting or non-counting.

How carbon in imported HWP affect emissions/removals estimation is complicated and not easily understood because emissions/removals associated with HWP are determined by the balance between inflow and outflow of carbon in HWP pool. For example, Japan applying the production approach with KP-LULUCF rule, reported HWP pool as net sink for recent three years, while as net source for most of other years. This is mainly due to the increasing share of domestic production in wood panels consumption, even if the amount of wood panels consumption itself has been decreasing over years. This situation leads domestic-origin carbon inflow in newly produced wood panels becomes larger than domestic-origin carbon outflow from the end-of-life wood panels, which had mainly produced from imported wood.

### Avoiding global double-counting of NDCs under the PA

Nearly two-thirds of countries include forests in their INDCs, but they account for 95% of global of round wood production. Forsell et al. [26] also assessed that the countries include LULUCF sector in their INDCs account for most of global net LULUC emissions in 2010 (based on FASTAT emission data which excluding HWP contribution). Grassi et al. [33] assessed the contribution of LULUCF in INDCs could provide about a quarter of total emission reductions planned in 2030 and also analyzed that majority of this LULUCF global emission reductions can be achieved by some large emitter of GHG in the LULUCF sector (Brazil, Indonesia and Russia).

The above-mentioned results indicate the current INDCs already cover most of global net emissions from the LULUCF, expected global emission reductions in the LULUCF and wood producing economy in the world, despite one-third of countries exclude forests in their INDCs.

Regarding the GHG quantification for HWP contribution to INDCs, 60% of global HWP contributions is included in INDCs, 20% is not properly assessed in INDCs, and remaining 20% is beyond the scope of GHG quantification for HWP in INDCs.

This means that for most countries, for whom forestry is a dominant part of their economy, have listed forestry in their INDCs and shown their intension to use the mitigation efforts related to HWP. However, some wood-producing and consuming countries still not reached the stage of quantifying their HWP contributions. This is considered one of the challenges in improving the contribution of HWP in global mitigation efforts in this sector.

The Katowice rule book on the mitigation accounting for NDC [24] did not provide a common accounting approach. But if a single globally applicable HWP approach to mitigation accounting is desired, the following situations should be considered: (1) ‘instantaneous oxidation’ is necessary for countries in which HWP is minor category (almost one third of the countries in the world are expected in this situation) and (2) ‘stock-change’ approach and ‘atmospheric-flow’ approach are not suitable for countries in which only a subset of forests is covered under their NDCs. Thus, a universal HWP “accounting” approach should combine ‘instantaneous oxidation’ and ‘production’ approach.

Trying to avoid global double-counting or non-counting of carbon in HWP may be futile when HWP is not completely covered in INDCs. Under the PA, the contribution must be “nationally determined” and so the accounting guidance for mitigation adopted by the Conference of the Parties serving as the meeting of the Parties to the PA (CMA1, November 2016) is considered to be a practical solution.

The IPCC guidelines provide three tiers how to estimate emissions/removals in each methodology: easier method using default parameters (Tier 1), more accurate method using country-specific parameters (Tier 2) and sophisticated method such as using a model (Tier 3). The tier chosen and the applied methods or models applied also affect the estimated result [34, 35]. More accurate estimates of HWP require proper data which may not be completely available at present [36]. The purpose of avoiding global double-counting or non-counting could be more fully understood by seeking accurate global estimations. If so, applying advanced methods with using better data is also important for more accurately assessing HWP in global level.

### HWP approaches suitable for REDD+

It is true that the use of harvested wood is relevant to the mitigation effects of REDD+ and that demand-side actions relating to wood are also necessary for implementing REDD+. At the same time, programs to reduce deforestation and/or forest degradation and increase wood usage are often implemented under different mitigation schemes. HWP estimation requires a different dataset in addition to a forest monitoring system. Therefore, a seemingly realistic solution would be to use ‘instantaneous oxidation’ for the REDD+ framework and include HWP mitigation actions under the INDC as necessary.

## Conclusion

Based on the GHG inventories, the carbon sequestration impact of HWP in Annex I countries was about 9.2% of the carbon sequestration in forest land, which contributed to offset about 1% of offset to the total net GHG emissions as an average for the period from 1990 to 2016. Two-thirds of the Annex I countries estimated that their HWP carbon pool increased during this period.

112 countries included forests in their INDCs and had a nearly 95% share of the global wood harvesting volume. Fifty-one of these countries include the impact of HWP in the emissions/removals estimations part of their INDCs and have a nearly 60% share of the global wood harvesting volume. In contrast, fifty-three countries do not include forests and HWP carbon pools in their INDCs and Seventy-two countries do not calculate the HWP contribution in estimations of emissions/removals for their INDCs.

All of this means that ‘instantaneous oxidation’ is necessary for estimating carbon stock changes in the HWP pools of countries where forest land is not a dominant land use category in order to avoid allocating too many resources because HWP is a minor category for them. In addition, for those countries that do not include total domestic forests and/or wood harvesting in their INDCs, ‘stock-change’ and ‘atmospheric-flow’ approaches cannot be used as a common accounting approach because the calculations for these approaches require carbon flows from both total domestic wood harvesting and total wood import/export data. For countries whose INDCs do not cover all domestic forest land and/or all wood harvesting, the captured carbon from domestic harvesting cannot be comprehensively calculated and so the total carbon stock changes derived from these calculations will not yield meaningful information.

In terms of the occurrence of global double-counting or non-counting of carbon in traded wood caused by combinations of different HWP approaches, it is necessary to consider not only differences in the system boundaries of the six HWP approaches but also differences between methods, pool-based vs. flux-based. Various combinations of HWP approaches can provide an overview for understanding whether double-counting or non-counting of carbon will occur but the same cannot be said about only the four most well-known approaches (‘instantaneous oxidation’, ‘stock-change’, ‘production’, and ‘atmospheric-flow’). If global double-counting or non-counting can be avoided by choosing the most appropriate HWP approach, then the combination of instantaneous oxidation with other approaches must be selected as the most pragmatic approach for some countries.

The decisions about the PA adopted at COP24 in Katowice suggested that parties use ‘production’ approach when estimating the HWP contribution to their GHG inventories under the guidance for GHG inventory of the PA but no uniform reporting/accounting approach for HWP was recommended in the context of NDC accounting.

The most pragmatic solution to the issue of determining a common HWP approach applicable to all countries would be to combine ‘instantaneous oxidation’ with approaches using the production system boundary (‘production’, ‘SCAD’, and/or ‘simple-decay’). This would be very similar to the approach currently taken under the guidance of the GHG inventory under the PA.

A drawback to this solution is that countries do not calculate in a consistent manner when the CO_2_ from HWP is released. This is due to the fact that ‘instantaneous oxidation’ estimates all subsequent emissions from HWP at the time of harvesting, whereas ‘production’ approach estimates when emissions from HWP actually occur. Previous studies [34, 35] have shown that the estimated amount of CO_2_ emissions and removals associated with HWP differ depending on which tier provided in the IPCC guidelines is applied, even when the same HWP approach is used. In addition, more accurate estimates require proper data that may not be completely available at present. Furthermore, the impact of double-counting or non-counting that occurs, especially at the accounting level, can be assumed to be less than the impact at the estimation level because the accounting amount is calculated by taking the difference between the baseline and the actual estimation, after which most of the double-counting of carbon is canceled out.

From the perspective of accuracy, it is worth establishing a common HWP approach that will not lead to global double-counting and non-counting. It is also important to improve the estimation methodologies of HWP at the national level.

## Methods

### Estimating forest land and HWP contributions to INDCs

The INDC classification is determined based on the following four elements. The first is whether or not forest land is included. This can be determined by reviewing the information on scope and categories/activities/policies covered in the INDC. The second is whether or not a forest listed in the NDC is considered in calculations for GHG emissions/removals. When the contribution of a forest related to a sector/category/activity is represented as a planted area, as a forest volume, or as a policy/measure rather than a GHG emission/removal amount, then it is not considered under GHG emissions/removals. The third is whether or not all forest harvesting is considered or could be included. When only part of a forest area or some forest-related activities are included in the INDC (e.g., including deforestation but excluding forest degradation and forest management), then only part of forest harvesting is considered to be covered under the INDC. The fourth is whether or not the applied IPCC guidelines allow for HWP contributions to be calculated using approaches other than ‘instantaneous oxidation’. This is determined whether or not the 2006 IPCC Guidelines are used. Under the 2006 IPCC Guidelines, the HWP contribution can be calculated using approaches other than ‘instantaneous oxidation’; however, under the Revised 1996 IPCC Guidelines or GPG-LULUCF, instantaneous oxidation must be used.

The assessment of forest harvesting volume for each classification in Table 2 is based on the volume of round wood production in 2017 according to FAOSTAT (ID# 1861) [26]. Each country’s share of the total global round wood production is calculated based on the totals for each INDC classification.

Some countries provide no information beyond the fact that REDD+ was used in their INDCs. As a result, the scope and coverage of their forests and how they treat forests when determining their INDCs is unknown. In order to clarify these details, additional analyses were conducted to determine the coverage of activities, carbon pools, and geographical area of these countries from the information in the submitted the reference level of REDD+ which are available at REDD+ platform [37], regardless of whether REDD + was used to determine the land-use sector’s contribution to their INDCs. Table 6 provides a summary of the scope of activities, carbon pools, and geographical boundaries in REDD+ for all countries based on technical assessment reports of the reference levels for the 2015–2018 assessment cycles and reference level submissions for the 2019 assessment cycle. Based on an analysis of coverage of activities, carbon pools, and geographical boundaries, only 12 of the 39 REDD+ countries included all forest harvesting in their reference levels; the harvesting coverage of the other 27 countries is not considered to be comprehensive. Only 1 of these countries included its HWP contribution when calculating REDD+.

**Table 6:** Summary of the scope of activities, carbon pools, and geographical boundaries in REDD + reference levels

Countries	Carbon pools included						Activities included					Boundary	Includes all harvesting
	AGB	BGB	DW	LT	SOC	HWP	Def	Deg	FC	SFM	Enh		
Argentina	X	X					X					Sub-N	
Bangladesh	X	X					X	X			X	N	X
Brazil	X	X	X	X			X					Sub-N	
Cambodia	X	X					X	X			X	N	X
Chili	X	X	X				X	X	X		X	Sub-N	
Columbia	X	X					X					Sub-N	
Congo	X	X	X				X	X				N	X
Costa Rica	X	X	X	X			X				X	N	
Cote d’Ivoire	X	X	X	X			X				X	N	
DRC	X	X					X					N	
Ecuador	X	X	X	X			X					N	
Ethiopia	X	X	X				X				X	N	
Ghana	X	X	X	X	X	X	X	X			X	N	X
Guinea-Bis	X	X					X				X	Sub-N	
Guyana	X	X	X				X	X				N	X
Honduras	X	X	X	X			X					N	
India	X	X	X	X	X					X		N	X
Indonesia	X				X		X	X				N	
Lao PDR	X	X					X	X			X	N	X
Madagascar	X	X	X		X		X					N	
Malaysia	X	X								X		Sub-N	
Mexico	X	X					X					N	
Mongolia	X	X	X	X			X	X			X	N	X
Mozambique	X	X					X					N	
Myanmar	X	X		X			X				X	N	
Nepal	X	X					X	X			X	N	
Nicaragua	X	X					X	X			X	N	
Nigeria	X	X					X					N	
Panama	X	X	X	X			X	X	X	X	X	N	X
PNG	X	X					X	X			X	N	X
Paraguay	X	X					X					N	
Peru	X	X					X					Sub-N	
Solomon Isl	X	X					X	X			X	N	X
Sri Lanka	X	X		X			X				X	N	
Suriname	X	X	X				X	X				N	X
Uganda	X	X					X					N	
Tanzania	X	X	X				X					N	
Vietnam	X	X					X	X			X	N	
Zambia	X	X	X				X					N	
Total	39	38	17	11	4	1	37	16	2	3	19	N:30	12

Based on the technical assessment reports of forest reference emission levels (FREL) for the 2015–2018 assessment cycles and the FREL submissions for the 2019 assessment cycle

*AGB* above ground biomass, *BGB* below ground biomass, *DW* dead wood, *LT* litter, *SOC* soil organic carbon, *HWP* harvested wood products, *Def* deforestation, *Deg* forest degradation, *FC* forest conservation, *SFM* sustainable forest management, *Enh* enhancement of forest removals, *N* national approach used, *Sub-N* subnational approach used

### Logical analysis of potential double-counting of each combination of HWP accounting approaches

A logical analysis was conducted to clarify the occurrence of double-counting or non-counting of emissions/removals associated with HWP among countries according to the HWP accounting approach used. In this analysis, the features of each HWP approach are differentiated based on how the transfer of carbon was treated between forest land carbon pools, HWP pools, and the atmosphere (Fig. 2, Table 2);

- On-site absorption: carbon sequestration by forest biomass.
- On-site emissions: all carbon releases to the atmosphere from forest sites (e.g., decomposition).
- Off-site emissions: all carbon releases to the atmosphere outside of forest sites except for the HWP pool, including emissions from feedstock or wood residue during processing.
- From forest land to HWP as domestically utilized wood: carbon transfer from domestic forest land pools to the domestic HWP pool for consumption.
- From forest land to exported HWP: carbon contained in exported HWP transferred from the producing country to the HWP pools of other countries.
- From forest land in other countries to imported HWP: carbon contained in imported HWP transferred from other countries to the domestic HWP pool for consumption.
- From HWP as domestically utilized wood to the atmosphere: carbon transfer from the domestic HWP pool to the atmosphere.
- From exported HWP to the atmosphere: carbon transfer from the HWP pool of exported HWP (i.e., used in another country) to the atmosphere.
- From imported HWP to the atmosphere: carbon transfer from the HWP pool of imported HWP (i.e., used domestically) to the atmosphere.

The summary of the treatment about the carbon transfers in each HWP approach is shown in Table 3.

### Analysis of current HWP contributions in reported GHG inventories

An assessment of HWP reporting was conducted based on national GHG inventories from Annex I countries containing their emissions and removals for the period from 1990 to 2016 [28]. The HWP approach used by each country was checked against National Inventory Report (NIR) information and the numbers reported in the common reporting format (CRF) tables. For some countries, the HWP approach used was not clearly explained in the NIR/CRF or reporting mistakes were found in the CRF. In such cases, the HWP approach was identified by referencing the method and data used as well as the background papers cited in the NIR.

Analyses of HWP trends were conducted and the ratio of HWP shares to forest land and to national total emissions was calculated by comparing the relevant reported emissions and removals reported in the CRF tables in the GHG inventories for each year. However, for Canada, the HWP contribution was not identical to the total carbon stock changes in forest land and HWP pools because the simple-decay approach was used. Thus, the change in carbon stock from the previous year was used as a proxy HWP contribution. The ratio of HWP pool share to forest land pools was calculated by dividing the total HWP carbon stock change by the total carbon stock changes in forest land and HWP. The share of the HWP contribution and total national emissions was calculated based on a CO_2_-equivalent basis. This analysis was conducted for total national emissions both including and excluding LULUCF, which are very common values used in the GHG inventory reporting.

In addition to the above factual basis analysis, the hypothetical potential contribution to the “accounted” emissions reduction volume that can be archived by HWP was considered. In this consideration, a hypothetical baseline was assumed to be the average of net emissions or removals of HWP for the period from 1990 to 2016. The hypothetical “actual” emissions are estimated from the top one-third of the largest net removals of HWP for the period from 1990 to 2016. Thus, the hypothetical results derived from the comparison of “actual” estimations and baselines only yields the carbon credit. This is based on the intention to determine the maximum potential HWP contribution to the emissions reduction volume.

## Data Availability

The data supporting our conclusions are available either in the paper itself or in the links listed in the references. Additional data may be requested from the corresponding author.

## Abbreviations

AR: Afforestation and Reforestation
AWG-KP: Ad-Hoc Working Group for the Kyoto Protocol
C: carbon
CMA: Conference of the Parties serving as the meeting of the Parties to the Paris Agreement
COP: Conference of the Parties
CRF: Common Reporting Format
FAO: Food and Agriculture Organization
FM: Forest Management
FREL: Forest Reference Emission Level
GHG: greenhouse gas
GPG: Good Practice Guidance
HWP: harvested wood product
INDC: Intended Nationally Determined Contribution
IPCC: Intergovernmental Panel on Climate Change
KP: Kyoto Protocol
KPSG: Revised Supplementary Methods and Good Practice Guidance Arising from the Kyoto Protocol
LULUCF: Land Use, Land Use Change and Forestry
MPGs: Modalities, Procedures and Guidelines
NIR: National Inventory Report
NDCs: Nationally determined contributions
REDD+: reducing emissions from deforestation and forest degradation and the role of conservation, sustainable management of forests and enhancement of forest carbon stocks in developing countries
SCAD: stock change approach from domestic origin
SWDS: Solid Waste Disposal Site
UNFCCC: United Nations Framework Convention on Climate Change

## Footnotes

1 Turkey reported its estimation of HWP in the cell of ‘stock-change’ approach in the CRF table but based on the information provided in its national inventory report and in a study by Bouyer and Serengil [29], the approach should be considered as a production approach.
